# O55 Polysaccharides Are Good Antigen Targets for the Formulation of Vaccines against O55 STEC and Capsulated aEPEC Strains

**DOI:** 10.3390/pathogens11080895

**Published:** 2022-08-09

**Authors:** Herbert Guimarães de Sousa Silva, Marcia Regina Franzolin, Geovana Ferreira dos Anjos, Angela Silva Barbosa, Luis Fernando dos Santos, Kaique Ferrari Miranda, Ronaldo Maciel Marques, Matilde Costa Lima de Souza, Roxane Maria Fontes Piazza, Marta de Oliveira Domingos

**Affiliations:** 1 Laboratório de Bacteriologia, Instituto Butantan, Avenida Vital Brasil, 1500, São Paulo CEP 05503-900, SP, Brazil; 2 Centro de Bacteriologia, Núcleo de Doenças Entéricas, Instituto Adolfo Lutz, Avenida Dr. Arnaldo, 355, São Paulo CEP 01246-000, SP, Brazil

**Keywords:** *E. coli*, O55, EPEC, STEC, O-antigen polysaccharides, complement, phagocytosis

## Abstract

The serogroup O55 of *E. coli* is composed of strains whose mechanisms of virulence are different from each other. Since the O55 polysaccharides are present in all *E. coli* O55 strains, and so are the polymers that compose the capsule of O55 atypical enteropathogenic *E. coli* (aEPEC), it was investigated whether anti-O55 antibodies were able to help the innate immune system to eliminate capsulated aEPEC and Shiga toxin-producing *E. coli* (STEC) belonging to the serogroup O55. The results demonstrate that the capsule of EPEC was able to inhibit the deposition of C3b on the bacterial surface and, as a consequence, their lysis by the alternative pathway of the complement system. However, in the presence of antibodies, the ability of the complement to lyse these pathogens was restored. It was also observed that macrophages were able to ingest EPEC and STEC, but they were only able to kill the ingested pathogens in the presence of antibodies. Anti-O55 antibodies were also able to inhibit aEPEC and STEC O55 adherence to human epithelial cells. In summary, the results demonstrated that the O55 polysaccharides have the potential to induce an effective humoral immune response against STEC and EPEC, indicating that they are good antigen targets to be used in vaccine formulations against these pathogens.

## 1. Introduction

After pneumonia, diarrhea is the second leading cause of child mortality around the world (WHO) [1]. Five thousand children under five years old die each year from diarrhea, making diarrheagenic *E. coli* the main bacterial etiologic agent [1]. 

There are six well-described pathotypes of diarrheagenic *E. coli*: typical and atypical enteropathogenic *E. coli* (tEPEC and aEPEC), enterotoxigenic *E. coli* (ETEC), typical and atypical enteroaggregative *E. coli* (tEAEC and aEAEC), enteroinvasive *E. coli* (EIEC), diffusely adherent *E. coli* (DAEC), and Shiga toxin-producing *E. coli* (STEC) [2]. It is important to mention that STEC has a subcategory denominated enterohaemorrhagic *E. coli* (EHEC), whose main difference from the other STEC strains is the presence of a pathogenicity island (PAI) named locus of enterocyte effacement (LEE) which is also present in EPEC [3].

*E. coli* is also classified into serogroups and serotypes based on the composition of their somatic O antigen (Serogroup) and flagellar (H) antigens (serotypes) [4]. There are 187 formally defined serogroups of *E. coli* [5]. However, only a few are responsible for the majority of diarrheal diseases, including outbreaks of bloody diarrhea and hemolytic uremic syndrome (HUS) [6]. In addition, some of these serogroups are emerging pathogens that can be encountered in more than one category of DEC [6,7]. 

A good example of these pathogens is *E. coli* O55, whose strains can be classified as enteropathogenic *E. coli* (EPEC) and Shiga toxin-producing *E. coli* (STEC) [4,6,7,8].

The variability encountered in their mechanisms of virulence makes these pathogens responsible for different clinical symptoms. For instance, as EPEC, they are responsible for acute diarrhea and infant death in developing countries, whereas as STEC, they are responsible for outbreaks of bloody diarrhea and hemolytic uremic syndrome (HUS) worldwide [9,10].

In addition, the potential of *E. coli* O55 strains to emerge as new virulent pathogens is well illustrated by the emergence of EHEC O157:H7 from an ancestral O55:H7 EPEC strain which acquired the ability to produce type II Shiga toxin [11,12].

The virulence of these pathogens can also be enhanced by the presence of a capsule composed of polysaccharides identical to the O-antigen polysaccharides present in their LPS molecules. Accordingly, this type of capsule is denominated O-antigen capsule and classified as G4C capsules (Group 4 capsules) [13].

It has been demonstrated that immune protection against Gram-negative bacteria, including *E. coli*, can be acquired by generating antibodies against their O antigen polysaccharides or capsular polysaccharides [14,15,16,17,18]. On the other hand, it has also been observed that antibodies against O-antigen polysaccharides are not effective against strains with phenotypic variability between themselves, even if they belong to the same serogroup [19].

Accordingly, to determine whether O55 polysaccharides could be considered good antigen targets for the generation of an effective humoral immune response against aEPEC and STEC O55 strains, the influence of anti-O55 antibodies on the clearance of these pathogens by the innate immune system was investigated. 

## 2. Results

### 2.1. Visualization of the Bacterial Polysaccharide Capsule

**Figure 1:**
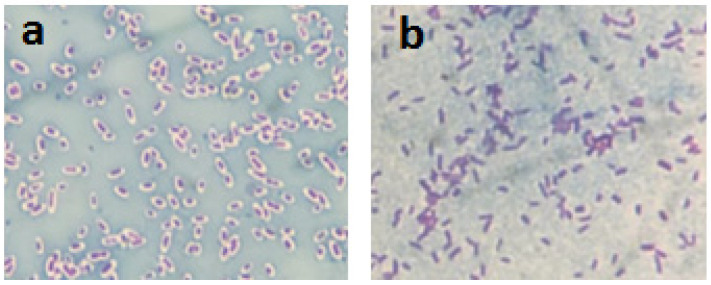
Visualization of the bacterial polysaccharide capsule—the presence of a capsule was determined by Maneval’s technique in aEPEC O55:H7 (**a**) and STEC O55:H19 (**b**) strains.

The results obtained from Maneval’s staining method demonstrate that the aEPEC O55:H7 strain is enveloped by a capsule, whereas the STEC O55:H19 strain is naked (Figure 1). 

### 2.2. Influence of Anti-O55 Antibodies in Bacterial Recognition and Complement Response

**Figure 2:**
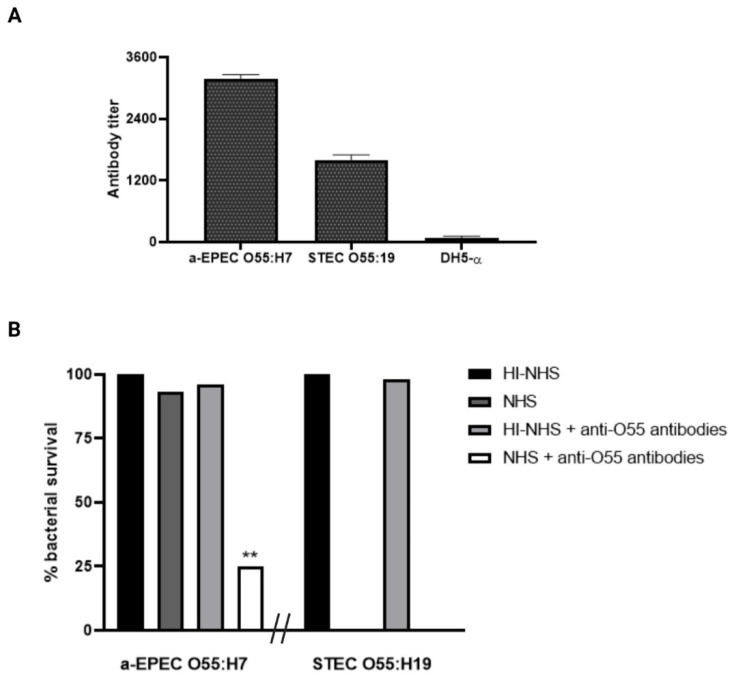
Influence of anti-O55 antibodies in bacterial recognition and complement response. (**A**) Antibody recognition. The ability of the antibodies against O55 polysaccharides to recognize a-EPEC O55:H7, STEC O55:H19, and DH5-α were determined by ELISA. (**B**) Bacterial lysis by the complement. The ability of the antibodies against O55 polysaccharides to help the complement system to lyse capsulated aEPEC O55:H7, STEC O55:H19, and DH5-α was determined by incubating the bacterial samples (10^12^) with rabbit serum against O55 polysaccharides (anti-O55 antibodies) in the presence of normal human serum (NHS) or heat-inactivated normal human serum (HI-NHS). NHS was used as a source of complement, and HI-NHS was used as a negative control for the complement action. The ability of the complement to lyse the bacteria was determined by counting the number of colony-forming units (CFU) after 3 h of incubation. ** (*p*-value ≤ 0.05) was considered statistically significant.

The ability of the antibodies against O55 polysaccharides to recognize capsulated aEPEC O55:H7 and noncapsulated STEC O55:H19 strains were analyzed by ELISA. The results demonstrate that the antibodies were able to recognize aEPEC O55:H7 and STEC O55:H19 but not a nonrelated *E. coli* strain (DH5-α) (Figure 2). It was also observed that the presence of anti-O55 polysaccharide antibodies was necessary to induce the lysis of capsulated aEPEC O55:H7 by the complement system (Figure 2B).

### 2.3. Deposition of C3b and C1q on the Bacterial Surface

**Figure 3:**
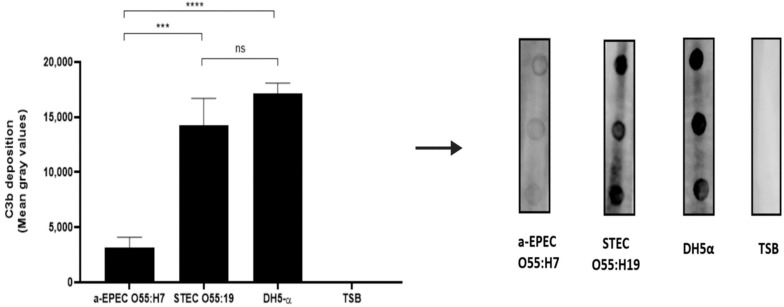
Deposition of C3b on the bacterial surface. C3b binding on the bacterial surface was determined by the dot blot technique. Nitrocellulose membrane coated with capsulated O55:H7 aEPEC, noncapsulated O55:H19 STEC, and noncapsulated DH5-α were incubated with normal human serum as a source of complement. The membrane was then incubated with goat IgG anti-C3b. After incubation, the membrane was washed and incubated with rabbit anti-goat IgG labeled with peroxidase. The deposition of C3b on the bacterial surface was detected by chemiluminescence (SuperSignalDensitometric) analyses, and the intensity of the signal was determined in pixels by Image J software developed at the National Institutes of Health and the laboratory for Optical and Computational Institutes (LOCI, University of Wisconsin, USA) Version 1.5. The results were plotted as “Mean Gray Values” (average of intensity units in selection). Unpaired *t*-test: ***/**** (*p*-value ≤ 0.05) was considered statistically significant. n.s. = not significant.

The dot blot technique was utilized to determine whether C3b deposition on the bacterial surface could be influenced by the presence of a capsule. The results demonstrated that the capsule present in aEPEC O55:H7 inhibited the deposition of C3b on the bacterial surface. In contrast, deposition of C3b was observed on the surface of naked STEC O55:H19 and DH5-α strains (Figure 3).

**Figure 4:**
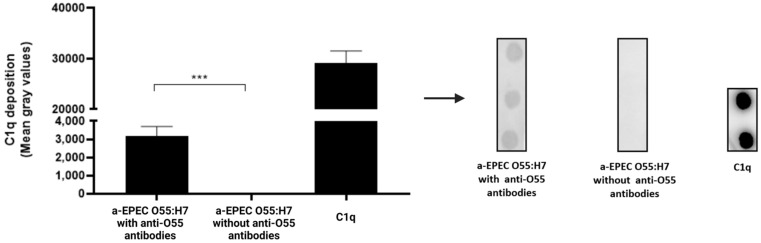
Deposition of C1q on capsulated aEPEC O55:H7 surface. For C1q determination, the nitrocellulose membrane was coated with capsulated EPEC O55:H7 previously incubated for 1 h at 37 °C in the presence or absence of anti-O55 antibodies. As a positive control, the membrane was coated with 125 ng of C1q. Subsequently, the membrane was blocked and incubated for 1 h at room temperature with 6.2 µg of C1q in PBS. The membrane was then washed and incubated with goat IgG anti-C1q. After incubation, the membrane was washed and incubated with rabbit anti-goat IgG labeled with peroxidase. The deposition of C1q on the bacterial surface was detected by chemiluminescence (SuperSignalDensitometric) analyses, and the intensity of the signals was determined in pixels by Image J software. The results were plotted as “Mean Gray Values” (average intensity units in selection). Unpaired *t*-test: *** (*p*-value ≤ 0.05) was considered statistically significant.

In order to determine whether the lyses of capsulated aEPEC O55:H7 in the presence of anti-O55 polysaccharide antibodies were related to the activation of the classical pathway of the complement system by antibody-opsonized capsulated aEPEC, the deposition of C1q on the surface of capsulated aEPEC O55:H7 was determined by the dot blot technique in the presence or absence of anti-O55 polysaccharides. The results demonstrate that deposition of C1q on the capsulated aEPEC O55:H7 surface was observed only in the presence of antibodies (Figure 4).

### 2.4. Influence of Anti-O55 Antibodies in Phagocytosis and Bacterial Adhesion

**Figure 5:**
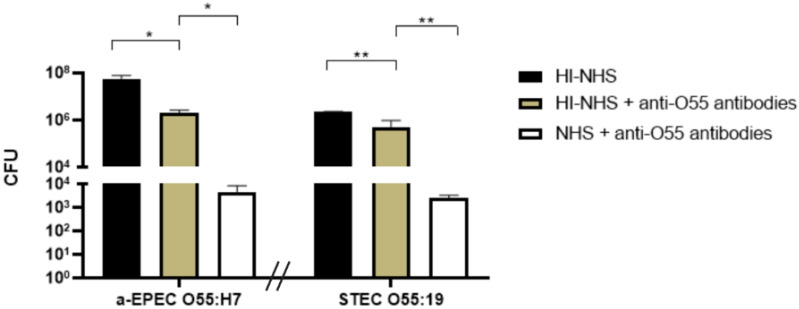
Influence of anti-O55 antibodies on the bacterial clearance by phagocytes. Macrophages were incubated with either capsulated aEPEC or STEC in the presence or absence of rabbit serum against O55 polysaccharides (anti-O55 antibodies). Normal human serum (NHS) was used as a source of complement, and heat-inactivated normal human serum (HI-NHS) was used as a negative control. After incubation, the phagocytes were lysed, and the amount of ingested bacteria was determined by counting the number of colony-forming units (CFU). Unpaired *t*-test: */** (*p*-value ≤ 0.05) was considered statistically significant.

The influence of antibodies against O55 polysaccharides in the phagocytosis and clearance of capsulated aEPEC O55:H7 and STEC O55:H19 was determined in macrophages. The results demonstrate that the phagocytes were able to ingest both aEPEc and STEC. However, the ability to kill the ingested pathogens was more efficiently achieved in the presence of antibodies, and normal human serum (NHS) was used as a source of complement (Figure 5). 

**Figure 6:**
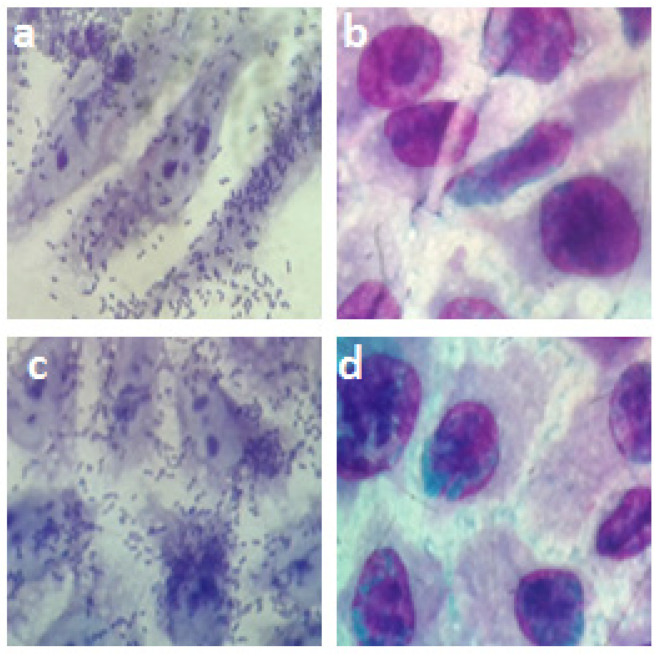
Influence of anti-O55 antibodies on bacterial adhesion. HEp-2 cells were incubated for 2 h with: (**a**) aEPEC O55:H7 in DMEM; (**b**) aEPEC O55:H7 in the presence of anti-O55 polysaccharide antibodies; (**c**) STEC O55:H19 in DMEM; (**d**) STEC O55:H19 in the presence of anti-O55 polysaccharide antibodies. An ocular 10 objective (×100) was used for visualization.

In regard to bacterial adhesion, the results showed that antibodies against O55 polysaccharides were able to inhibit the adherence of aEPEC O55:H7 and STEC O55:H19 to the epithelial cells (Figure 6).

## 3. Discussion

Bacterial capsules are composed of diverse repeat units of polysaccharides whose basic function is to protect the cell against host defenses, grazing, and environmental stress [13,20,21]. They also play a significant role in virulence since they have acquired different mechanisms of immune evasion during evolution, especially against lysis by the complement system [22,23].

One of the mechanisms used by bacteria to avoid being killed by the complement system is well illustrated by k1-capsulated extraintestinal *E. coli* (ExPEC), whose capsule activates the FH factor—a negative complement regulatory protein that downregulates the alternative pathway of complement [24]. 

In contrast, the capsule of aEPEC O55:H7 impaired the activation of the alternative route of the complement system by inhibiting the deposition of C3b on the bacterial surface. The lytic activity of the complement system, however, was restored in the presence of anti-O55 antibodies, indicating that the classical route of the complement system was activated, as suggested by the deposition of C1q only on the surface of antibody-opsonized aEPEC. This result emphasizes the importance of antibodies on the clearance of O-antigen capsulated *E. coli* strains, especially those of septicemic serogroups, whose capsule is essential for serum survival and multiplication in the bloodstream [23].

Phagocytosis, another crucial process involved in the clearance of pathogens, can also be enhanced by the presence of antibodies and complements [25]. The cooperation between these opsonins is demonstrated herein by the increased clearance of ingested aEPEC O55:H7 and STEC O55:H19 strains in the presence of both antibodies and complements. This finding shows the importance of antibodies in the process of elimination of capsulated pathogens such as aEPEC and *E. coli* strains that can produce antiphagocytic factors such as STEC [26,27].

An additional aspect related to STEC is the fact that antibodies are not indicated as a treatment against these pathogens since antimicrobial molecules can stimulate the release of Shiga toxins by them, consequently increasing the patient’s risk of developing hemolytic uremic syndrome (HUS) and acute renal failure. As a result, the management of HUS mainly involves supportive care [28,29,30]. Accordingly, several groups have been working with the aim to develop alternative treatments, such as the use of monoclonal antibodies to neutralize the action of Shiga toxins and the development of vaccines to prevent infection [31,32,33]. 

In relation to the development of vaccines against diarrheagenic *E. coli*, it should be taken into consideration that bacterial adhesion is a critical step that precedes colonization and infection. Therefore, an effective vaccine against STEC and other diarrheagenic *E. coli* strains has to generate antibodies that can inhibit the adhesion of these pathogens to the intestinal cells [31,32]. However, the variability encountered between the mechanisms of virulence of diarrheagenic *E. coli*, including strains that belong to the same serogroup, makes the development of vaccines against these pathogens a challenging task [4,5,6,7,8,9,10,11,12,13,14,15,16,17,18,19,20,21,22,23,24,25,26,27,28,29,30,31,32,33,34]. Nonetheless, in the present work, it has been demonstrated that antibodies against O55 polysaccharides are able to inhibit the adhesion of two different categories of diarrheagenic *E. coli* (aEPEC and STEC) to epithelial cells. This result indicates that the generation of antibodies against O55 polysaccharides in the intestinal mucosa has the potential to prevent the adhesion and subsequent colonization of the gut by aEPEC and STEC belonging to the serogroup O55.

In summary, the results presented herein suggest that the use of O55 polysaccharides as antigen targets could be valuable for the development of vaccines against aEPEC and STEC belonging to the serogroup O55.

## 4. Material and Methods

### 4.1. Bacterial Strains

The strains used in this study were O55:H7 (aEPEC) [35], O55:H19 (STEC) [36], and *E. coli* K12 DH5α purchased from Bethesda Research Laboratories (Maryland, NJ, USA). All the strains were derived from the *E. coli* collection of the Laboratory of Bacteriology of the Instituto Butantan, São Paulo, Brazil. 

### 4.2. Cell Lines

The HEp-2 and J774A.1 cell lines used in this study were obtained from the Instituto Adolfo Lutz, São Paulo, Brazil. The cell lines were previously acquired from the American Type Culture Collection (CCL 2). For maintenance, HEp-2 cells were grown in high glucose—Dulbecco’s Modified Eagle Medium (DMEM), supplemented with 10% calf serum, 1 mM of L-glutamine, and 50 IU/mL of penicillin-streptomycin. J774A.1 cells were grown in RPMI 1640 (Roswell Park Memorial Institute) supplemented with 10% calf serum, 1 mM of L-glutamine, and 50 IU/mL of penicillin-streptomycin.

### 4.3. Anti-O55 Antibodies

Rabbit serum against O55 polysaccharides was obtained commercially from PROBAC (São Paulo, SP, Brazil). 

### 4.4. Capsule Visualization

The presence of a capsule was determined by Maneval’s method [37]. Briefly, bacterial cells scraped from a fully grown agar plate were mixed on a glass microscope slide with a 100 µL aqueous solution of 1% Congo red stain (Sigma-Aldrich, St. Louis, MO, USA). This suspension was spread across the microscope slide to form a thin film, which was air-dried. Approximately 5 mL of Maneval stain [3.33% phenol, 4.44% glacial acetic acid, 2.67% ferric chloride, and 0.02% acid fuchsin (Sigma-Aldrich)] was distributed on the slide and incubated for 2 min at room temperature. For visualization, the slide was drained and air-dried. The capsules were not stained and appeared white underneath the light microscope (eyepiece, ×10; objective, ×100).

### 4.5. Anti-O55 Antibody Detection

Antibodies were detected by enzyme-linked immunosorbent assay (ELISA). Briefly, the following bacterial cultures: aEPEC O55:H7, STEC O55:H19, and *E. coli* K12 DH5α were grown in Tryptic Soy Broth (TSB) for 18 h at 37 °C. Subsequently, the bacterial cultures were diluted 1/10 in TSB, added individually to 96 well cell culture plates, and incubated for 18 h at 37 °C. After incubation, the plates were washed with PBS, and the bacteria adherent to the plates were fixed with 75% ethyl alcohol for 1 h at room temperature. Next, the plates were washed three times with PBS containing 0.05% Tween 20 and incubated for 2 h at 37 °C in blocking buffer (3% BSA in PBS). After washing, two-fold dilutions of rabbit serum anti-O55 polysaccharides prepared in incubation buffer (1% BSA in PBS) were added in triplicate to the plates (starting dilution 1/100). The plates were then incubated for 2 h at 37 °C. Subsequently, the plates were washed and incubated for 1 h at 37 °C with anti-mouse IgG-alkaline phosphate conjugate diluted 1/2500 in incubation buffer. The plates were washed once more, and then the enzymatic reaction was developed with 5 mg/mL of p-nitrophenyl phosphate in diethanolamine buffer (0.1 mL/well). The optical density was read at 405 nm in a Titertek plate reader after 15 and 30 min of incubation at room temperature. The titer was determined as the last serum dilution whose optical density was two-fold the value obtained with the negative control (*E. coli* K12 DH5α) at the dilution of 1/100.

### 4.6. Complement Lysis

In order to determine the ability of the complement system to lyse the bacterial samples, 5 µL of aEPEC O55:H7 and STEC O55:H7 (1 × 10^12^ CFU/mL) were added to a 96 well culture plate. The plate was then incubated for 16 h at 37 °C with 50 µL of rabbit anti-O55 serum in the presence of either 15 µL of normal human serum (NHS) or 15 µL of heat-inactivated normal human serum (HI-NHS). NHS was used as a source of complement. Subsequently, the bacterial viability was determined by counting the number of colony-forming units (CFU) according to the methodology described by Baron and coworkers and Beck et al. [38,39].

### 4.7. C3b Deposition on the Bacterial Surface

The deposition of C3b on the surface of aEPEC O55:H7 and STEC O55:H7 was determined by the dot blot technique. Briefly, nitrocellulose membranes (0.42 µM) were coated with 2 µL of bacterial samples (1 × 10^13^ CFU/mL). Subsequently, the membranes were incubated with 3% BSA in PBS overnight at room temperature. After incubation, the membranes were washed 3 times with PBS containing 0.05% Tween 20 (washing buffer) and incubated for 1 h at 37 °C with a solution of 10% normal human serum (NHS) in incubation buffer (1% BSA in PBS). NHS was used as a source of C3b. In sequence, the membranes were washed and incubated for 1 h with goat anti-C3b antibodies (Complement Technology, Tyler, TX, USA) diluted 1:5000 in incubation buffer. After incubation, the membranes were washed and incubated for 1 h with anti-goat IgG peroxidase conjugate (Sigma-Aldrich) diluted 1:10,000 in incubation buffer. The membranes were then washed, and the deposition of C3b on the bacterial surface was determined by chemiluminescence analyses utilizing SuperSignal West Pico Enhanced Chemiluminescent Substrate (Pierce Biotechnology, Inc.—Thermo Fisher Scientific, Waltham, MA, USA). The intensity of the signals was determined in pixels using Image J software. The results were plotted as “Mean Gray Values” (average intensity units in selection).

### 4.8. C1q Deposition on the Bacterial Surface

The C1q deposition on the aEPEC O55:H7 surface was determined by the dot blot technique. Nitrocellulose membranes (0.42 µM) were briefly coated in triplicate with 2 µL of aEPEC O55:H7 (1 × 10^13^ CFU/mL) culture previously incubated for one hour at 37 °C in the presence or absence of rabbit serum against O55 polysaccharides diluted 1/10 in PBS. Two microliters of C1q (125 ng/mL) were coated on the membrane to be used as a positive control. The membranes were then incubated overnight at room temperature in a solution of 3% BSA in PBS. In sequence, the membranes were washed 3 times with PBS containing 0.05% Tween 20 and incubated for 1 h at 37 °C with 6.2 µg/mL of purified C1q (Complement Technology) in incubation buffer (1% BSA in PBS). Subsequently, the membranes were washed and incubated for 1 h with goat IgG anti-C1q (Complement Technology) diluted 1:5000 in incubation buffer. After washing, the membranes were washed again and incubated for 1 h at room temperature with rabbit anti-goat IgG conjugated with peroxidase (Sigma-Aldrich) diluted 1:10,000 in incubation buffer. The membranes were then washed, and the deposition of C1q on the bacterial surface was determined by chemiluminescence analyses utilizing SuperSignal^®^ West Pico Enhanced Chemiluminescent Substrate (Pierce). The intensity of the signals was determined in pixels using Image J software. The results were plotted as “Mean Gray Values” (average intensity units in selection)

### 4.9. Phagocytosis

J774A.1 macrophages at a concentration of 5 × 10^4^ cells/mL in RPMI with 10% fetal bovine serum were seeded in 24-well cell culture plates (1 mL/well) and incubated for 48 h at 37 °C in a 5% CO_2_ incubator. In parallel, 20 µL of aEPEC O55:H7 or STEC O55:H19 (10^12^ CFU/mL) were incubated for 1 h at 37 °C with rabbit anti-O55 serum diluted 1/10 in RPMI containing 15% of normal human serum (NHS) or heat-inactivated normal human serum (HI-NHS). NHS was used as a source of complement. After incubation, the samples were added in triplicate to the cells and incubated for 3 h at 37 °C. The wells were then washed 3 times with PBS and incubated for 1 h with a solution of 10 µg/mL of ampicillin in PBS. Subsequently, the plates were washed 6 times with PBS, and the macrophages were lysed by incubating each well with a solution of 0.1% Triton X-100 in PBS (0.5 mL/well) for 10 min at room temperature. In sequence, 0.5 mL of PBS was added to each well, and the lysate was resuspended. The lysates were then submitted to a 10-fold serial dilution in saline, starting with a dilution of 1 in 10. In sequence, 10 µL of each dilution were added in triplicate to tryptic soy agar (TSA) plates to determine the number of colony-forming units (CFU) according to the methodology described by Baron and coworkers and Beck et al. [23,24].

### 4.10. Inhibition of Bacterial Adhesion to Epithelial Cells

HEp-2 cells were grown to 70% confluence on circular coverslips in wells of 24-well tissue culture plates in the presence of DMEM without antibiotics. Forty microliters of bacterial culture (aEPEC 055:H7 or STEC O55:H19) at a concentration of 10^7^/mL previously incubated for 1 h at 37 °C with rabbit serum against O55 polysaccharides diluted 1/2 in DMEM containing 1% fetal bovine serum were added in triplicate to the wells (1 mL/well) and incubated for 3 h at 37 °C in 5% CO_2_. As a positive control for bacterial adhesion, the cells were incubated only with bacteria in the absence of antibodies. After incubation, the monolayers were washed 6 times with sterile PBS and then fixed with 100% methanol for 10 min, stained for 5 min with May–Grunwald stain diluted 1:2 in Sorensen buffer, and finally stained for 20 min with Giemsa stain diluted 1:3 in Sorensen buffer. The excess stain was discarded, and the coverslips with the stained cells were affixed to microscope slides for visualization by light microscopy (eyepiece, ×10; objective, ×100). 

### 4.11. Statistical Analysis

Statistical analysis was performed with GraphPad Prism version 8.0.2, GraphPad Software, San Diego, CA, USA) employing the unpaired *t*-test. A *p*-value ≤ 0.05 (*) was considered statistically significant.

## Data Availability

The data presented in this study are available on request from the corresponding author. The data are not available in the repository of the Butantan Institute (https://repositorio.butantan.gov.br (accessed on 31 March 2022)).
